# Synthesis and Technology of Nanoemulsion-Based Pesticide Formulation

**DOI:** 10.3390/nano10081608

**Published:** 2020-08-17

**Authors:** Isshadiba Faikah Mustafa, Mohd Zobir Hussein

**Affiliations:** Materials Synthesis and Characterization Laboratory, Institute of Advanced Technology, Universiti Putra Malaysia, Seri Kembangan 43400, Selangor, Malaysia; isshyka1202@gmail.com

**Keywords:** nanoemulsion, agrochemical, properties, pesticide, agriculture

## Abstract

Declines in crop yield due to pests and diseases require the development of safe, green and eco-friendly pesticide formulations. A major problem faced by the agricultural industry is the use of conventional agrochemicals that contribute broad-spectrum effects towards the environment and organisms. As a result of this issue, researchers are currently developing various pesticide formulations using different nanotechnology approaches. The progress and opportunities in developing nanoemulsions as carriers for plant protection or nanodelivery systems for agrochemicals in agricultural practice have been the subject of intense research. New unique chemical and biologic properties have resulted in a promising pesticide nanoformulations for crop protection. These innovations—particularly the nanoemulsion-based agrochemicals—are capable of enhancing the solubility of active ingredients, improving agrochemical bioavailability, and improving stability and wettability properties during the application, thus resulting in better efficacy for pest control and treatment. All of these—together with various preparation methods towards a greener and environmentally friendly agrochemicals—are also discussed and summarized in this review.

## 1. Introduction

Colloidal nanoformulations have remarkable properties that have attracted much attention for use in various applications. These advanced nanoformulation systems bring many improvements in the agricultural sector for better efficacy towards antifungal activities [1,2] and pesticide delivery systems [3,4]. The incorporation of colloid nanoformulations into cosmetic products has enabled the modification of the drug permeation and allowing optimum efficiency on the skin [5,6]. This nanoformulation system also used for the food industry [7] to extend the shelf life of food [8] and improve food protection from the biodeterioration process [9].

Recently, the advantages of colloidal nanoformulation have opened up some alluring possibilities for enhancing technology in the agricultural sector. Industrial agriculture estimates that more than 70% of traditional pesticides are not efficient due to repetitive use at a higher dose in achieving optimal bio-efficiency thus leading to environmental fate through some process including runoff, leaching and volatilization [10].

Some of the conventional agrochemicals that are usually in emulsifiable concentrate or wettable powder forms are embedded in soil or groundwater for years. The long degradation period has resulted in its accumulation in the food chain and hazardous to the human and animal health and the environment. These pesticides have been banned through strict pesticide regulations in developed countries, but unfortunately, it is still widely used in many developing countries [11]. Hence, water-based pesticide formulations using colloidal systems can be a good alternative in replacing the existing formulation [12].

**Figure 1:**
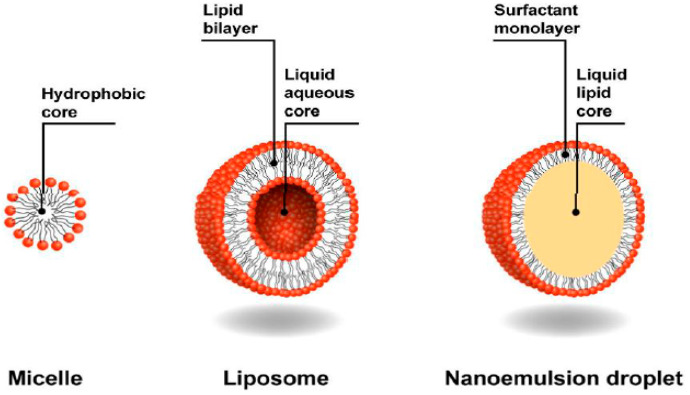
Composition of the micelle, liposome and nanoemulsion droplet (micelles with a hydrophobic core which is formed by the tails of the surfactant molecules. Liposomes with an aqueous core surrounded by a double phospholipid layer. Nanoemulsions droplets with a hydrophobic liquid core composed of the oil that is dispersed in the water and stabilized by a surfactant monolayer) Reproduced with permission from [14].

Figure 1 displays the type of colloidal systems: micelle, liposome and nanoemulsion which were developed to overcome the drawback of the conventional agrochemicals. Micelle is the term frequently used when emulsions are discussed. Microemulsion requires a high concentration of surfactant of 20% or more compared to only 3%–10% for nanoemulsion [13]. The development of nanoemulsion has many significant advantages as it reduces the use of organic solvents [12] or bioactive oil concentration and increases the solubilization of active ingredients while maintaining biologic activity. Along with the formation of nanoemulsion, spontaneous formation of micelle or the so-called micellization has also occurred, which indicates that micelle formation is to exist in the nanoemulsion.

Here, we discuss the synthesis, physicochemical and biologic characterizations and the technology of nanoemulsion-based pesticide formulation for agricultural use. The role of each component used for the formulation is described in detail. Additionally, studies of the use of the nanoemulsion for pesticide delivery in vitro and in vivo applications also are reviewed. Moreover, the penetration pathways of nanoemulsion-based agrochemicals and their active release into the living organism also are explained. The environmental risk assessment is also briefly indicated in this review.

## 2. Nanoemulsion as A Colloidal System

Nanoemulsion is also known as a miniemulsion, sub-micron emulsion or ultrafine emulsion, in which the size is between 20–500 nm [15]. The nanoemulsion structure can be custom-made to meet the needs of various applications. There are three types of nanoemulsion: oil in water (O/W), water in oil (W/O) and bi-continuous. In the later, the system is obtained when the oil and aqueous phase are separated by the surfactant layer. Nanoemulsions consist of three main parts: oil, surfactant and water. Two immiscible phases—oil or organic and water phase that are present in a nanoemulsion system—are separated by interfacial tension induces by surfactants [16].

### 2.1. Surfactant as an Emulsifier in Nanoemulsion

One of the important components in nanoemulsions are surfactants, also called emulsifiers. There are four types of surfactants: cationic, anionic, amphoteric and nonionic. In formulating nanoemulsion-based for pesticide applications, nonionic surfactants are usually encapsulated into the nanoemulsion, as they are less affected by the pH and ionic strength. This additional component can alter the stability and size of the nanoemulsion, as a result of cohesion between the anionic surfactant and the solution. The selection of surfactants also can be related to its hydrophilic–lipophilic balance (HLB) value. A higher value of HLB indicates an increase in surfactant solubility towards the water which favors by O/W formulations for pesticide formulations. The common value of HLB used to produce good O/W formulation in agriculture is in the range 10–16, as HLB value < 10 is usually considered as oil-soluble surfactants. In generating kinetically stable nanoemulsions, the HLB value of the surfactant is one of the most important parameters to be considered. A wide range of HLB values can be achieved either by single or mixed surfactants.

For example, a nanoemulsion against *Aedes aegypti* has been developed separately based on andiroba and copaiba oils with required hydrophilic–lipophilic balance (rHLB) values of 11.2 and 14.8. The nanoemulsions were found to be stable when the HLB value of surfactants was near to the rHLB oil in the system [17,18]. This study has strongly confirmed that when the HLB value of surfactants is similar to the rHLB of the oil used in the system, the most stable nanoemulsion was produced.

The incorporation of surfactant is usually between 1.5–10% and 5% is the most generally reported as appropriate and sufficient amount for nanoemulsion production. The use of a surfactant is believed to alter the electrostatic charge in the nanoemulsion which causes low aggregation [19]. The influence of single and complex surfactants in nanoemulsion formation has been studied previously [20]. Some studies have demonstrated mixed surfactant is capable to produce better hydrophilic–lipophilic balance (HLB), enhances the flexibility of the surfactant layer and ability to partition at high levels into the oil–water interface. A study also showed that a mixture of nonionic surfactants not only produced desired HLB value, but also gave a synergistic effect towards emulsion stability [21]. Table 1 presents a list of a single (non-ionic) and complex (anionic and non-ionic) surfactants that are used in the preparation of pesticide nanoemulsions.

The presence of surfactants has reduced the contact angle and increase the wettability of leaves. It has shown that a higher concentration of surfactants applied to pesticides does not promise a better contact angle on the adaxial and abaxial surface of the leaves. The optimum wetted area and contact angle may vary due to the nature of the surfactants [22]. The optimal surfactant concentration can give the desired particle size, stability, viscosity properties and antimicrobial activities that are needed for targeted useful applications. However, at a very high concentration, an excess surfactant may cause toxic effects. For better interaction on negatively charged plant leaves, ionic liquids (ILs) have been identified as additional surface-active agents for producing positive-charge nanoemulsion systems. The adsorption mechanism of leaves will tune the properties of nanoemulsion for better applications [23].

### 2.2. Oil as an Organic Phase and Carrier for Active Ingredients

Oils can be classified either as essential oils or non-essential oils. Essential oils (EOs) are not true oils; they are concentrated and volatile compound that usually comes from different parts of plant materials. They are known with their effectiveness, biodegradable and eco-friendly properties. In contrast, non-essential oils refer to a carrier oil that does not evaporate. The broad use of nanoemulsions as carriers of EOs has been widely reported as they reduce the volatilization [34], boost the bioactivity of EOs and reduce degradation processes such as oxidation, isomerization and polymerization [35]. Table 2 compiles a list of dispersant compositions that have been reported for the production of pesticide nanoemulsion.

A proper selection of oil as an oily phase has become one of the crucial parts for the formation of a nanoemulsion, as it affects the solubility of the active ingredient and facilitation of nanoemulsion formulation of the desired purposes. This selection step should be taken properly as it affects the further selection of other components in nanoemulsions, especially in the O/W nanoemulsion systems [36].

When peanut oil was used as the oil phase, it was found that the formation of the nanoemulsion is more difficult compared to a long-chain oil, such as hexadecane. The insolubility of oil in the system also increases the stability of the nanoemulsion by providing a kinetic barrier to the Ostwald ripening. Ostwald ripening is the net transport of oil at smaller droplets to larger droplets through continuous phase [37]. The dissolution of oil in nanoemulsion could enhance the cytotoxicity, genotoxicity and antimicrobial activity against pathogens as the oil constituents are rich with biologic properties. Different phytochemical composition in essential oils will affect the biologic behaviors towards the pathogens [38].

Recently, it was identified that *Aniba* essential oils disrupted the cell membrane of all eight phytopathogen types including *Aspergillus flavus, Aspergillus niger and Fusarium solani* by triggering more nucleic acid and protein productions [39]. *Vitex Negundo* essential oil is introduced to fight against *Avena fatua* and *Echinochloa crus-galli* weeds. *Asteriscus graveolens* oil can inhibit *Fusarium oxysporum* fungi which cause the Bayoud disease in date palm [40]. The finding proves the *V. negundo* EOs is toxic to weeds by reducing their mitotic index (MI) and chromosomal aberration percentage [41].

EOs are also known for use in controlling insects due to their volatility properties, which makes them suitable for fumigation [42]. Some authors demonstrated the increase of jojoba oil concentration causes the rise of death in rice weevil, *Sitophilus oryzae* adults [43]; the mortality rate of mosquito larvae also increases when surfactants are used higher in neem oil nanoemulsions and *Citrus sinensis* nanoemulsions, respectively [44,45]. Therefore, it was concluded that the mortality rate of pest is dependent on both surfactant and oil concentration in the nanoemulsions.

### 2.3. Solvent as an Aqueous Phase in the System

A variety of solvents are used in formulating nanoemulsion systems. The use of solvents can improve the properties of aqueous phase (viscosity, density, interfacial tension) and structural properties of surfactant solutions include optimum curvature and critical micelle concentration [60]. In recent years, attention has been focused on the use of a green solvent in replacing conventional organic solvents to achieve more environmentally pesticide nanoformulations. Some solvents such as N, N-methyl oleate [61], dimethyldecanamide (AMD-10) and D-limonene, [29] are considered as bio solvent-safe for agrochemical industry which leading to low risk for farmer or customers.

## 3. Role of Nanoemulsion in Pesticide Formulations

Pesticide nanoemulsion formulations are formulations in which active chemicals used in treating or preventing the crops from any disease which affects agricultural yield have been incorporated into the nanoemulsion system. These types of pesticides have been categorized based on their target organisms. The common pesticide formulations are active chemicals which are able to kill fungi (fungicides), kill weeds (herbicides), kill insects such as snails and slug (insecticides), etc. In achieving the maximal efficiency of pesticide delivery, the nanoemulsion acts as a vector that carries and delivers the bioactive compounds, the agrochemical to the target pests in the plants [12]. Their attractive physicochemical properties such as tunable nanosize have resulted in a larger surface area, thus enabling the release, accumulation and uptake of the active ingredients more effectively compared to their counterparts. The incorporation of the active ingredient into the nanoemulsion formulations has contributed to better kinetic stability [62], enhances the solubility and dissolution of poorly water-soluble agrochemicals, low surface tension and good wettability which conduce to highly improved foliage adhesion with which the pesticide will able to stay longer on leaves or other essential parts of the plant [28]. The nanoemulsion also can act as a coating layer for pesticides, providing greater protection against photodegradation [63].

## 4. Fabrication of Nanoemulsion in Agricultural Applications

Various methods have been developed and modified to produce nanoemulsion structures with stable conditions to suit the target application. Most of the pesticides based nanoemulsions are formed as O/W formulations. This nanoemulsion is preferable in improvising the dissolution absorption of agrochemicals as they are hydrophobic. Previous works revealed that the addition of polymer aided hydrophobicity groups, hydrophobically modified polyacrylamide resulted in a better effect on O/W nanoemulsion stability compared to the hydrolyzed polymer. The presence of a compound with hydrophobicity groups has enhanced the electrostatic interactions on droplets interface thus improving properties of nanoemulsion significantly [64]. It is also notable that the concentration of polymer used in nanoemulsion can affect the resulting properties of the nanoemulsion [65].

### 4.1. Preparation Method of Pesticide-Based Nanoemulsions

**Figure 2:**
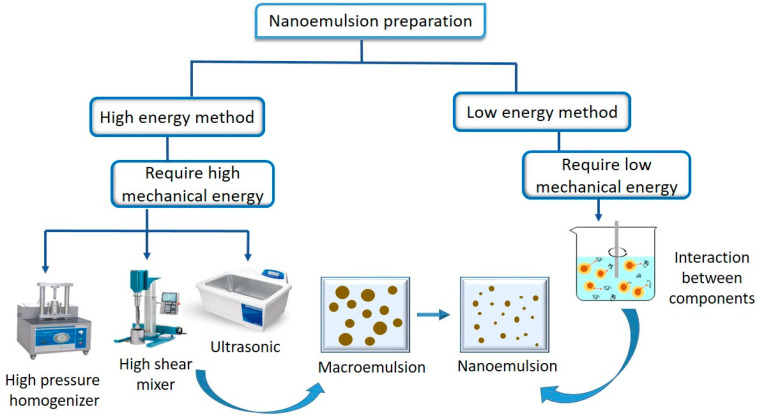
Schematic diagram showing a comparison between the high and the low-energy method, in which the former requires a specific device to break macroemulsion into nanoemulsion compared to the low-energy method, in which the energy is gained through the interaction of the components during the emulsification process.

High energy is necessary to initiate stress level to get above Laplace pressure with a pressure 10–100 atm for the transformation of nanoemulsion into stable systems by rupturing the droplets. The energy applied can be either a high-energy method or a low-energy method, as depicted in Figure 2.

#### 4.1.1. Low-Energy Method

The low-energy method is designated as the internal interaction of components in systems which dependent on surfactants behavior during the emulsification process. There are many studies in which this technique was adopted for the preparation of nanoemulsion [28,30,52,58]. This method includes phase inversion and self-emulsifying methods [66]. The spontaneous or so-called self-emulsification method is a component interaction initiated by the rapid diffusion of solvent or surfactants without changes in surfactant curvature in the system. This method can be accomplished by the aqueous phase or oil phase titration process. The concentration of the oil phase and surfactants are varied during the preparation and ternary phase diagram based on three components: surfactant, water and oil are generated [67]. The isotropic region shown by the ternary diagram indicates various combinations of formulations. All nanoemulsion regions in the phase diagram are considered as the optimum formulation with minimum surfactant concentration is selected. The selected formula is used thoroughly in developing nanoemulsion for further study.

Once there are changes in surfactant curvature during the emulsifying process, the method is called the phase inversion method. The phase transitions are induced based on two factors, either temperature or composition, thus known as phase inversion temperature (PIT) and phase inversion composition (PIC), respectively. Any surfactant can be applied in the PIC method compared to the PIT method, which only accessible for the surfactants which sensitive to temperature such as polyoxyethylene-type surfactants as reported previously [68]. PIT method is more advantageous than the self-emulsifying method as the organic solvent can be exempted from the former [69].

In addition, there is another modified method, so-called in situ phase inversion. This technique is conducted by mixing oil, surfactant and solvent simultaneously without any equipment needed. Since there is no strong forces applied, adding extra emulsifier into the aqueous phase is encouraged to obtain small nanoemulsion size [28]. Another green approach has also been suggested, the so-called solvent-free low-energy method. This method has less impact on the environment and can be operated at a lower cost. The non-heating process also was suggested where essential oil has been used in the nanoemulsion formulation. Recently, this method has been used to develop nanoemulsions in controlling *Aedes aegypti* mortality [18,70].

#### 4.1.2. High-Energy Method

This method requires a device to generate intense forces to produce smaller formulations. High shear stirring, ultrasonication and high-pressure homogenization are some techniques adopted under this method. The amount of energy input is inversely proportional to the size of the nanoemulsion.

Among the high-energy methods, ultrasonication is the easiest method used in fabricating nanoemulsions. These strong disruptive forces produced by the ultrasonic processor cause extreme shear which breaks up droplets to produce nanoemulsions. Higher sonication time creates more kinetic energy to the emulsion and imparts the reduction of particle size [71]. Ultra-Turrax homogenizer is a mechanical device used in the high-pressure method. The applied energy forces a liquid through a specified valve under high pressure to produce high-speed impact leading to droplet rupture before good dispersion is generated. High shear stirring is referring to the shear forces produced by the velocity of droplet flow at a small gap between rotor and stator. Due to simple operation and low power consumption, the high shear stirring method becomes more favorable than the high-pressure method. This high-energy approach for the preparation of nanoemulsion has been previously described elsewhere [46,47,48,49,50].

The stability of nanoemulsion-based agrochemicals is also related to the sequence of the steps taken during the initial preparation. All preformulation process involves two phases: organic and aqueous phase. Normally, the oil phase consists of oil and dispersant while the aqueous phase is a mixture of surfactants with water. In a previous study, the addition sequence of organic and aqueous phases are altered to obtain maximal stability [61]. The study identified that the addition of the aqueous phase into the organic phase has contributed to the highest emulsion stability. This is in line with the result of other researchers [72]. Emulsification treatment at the final step also plays a big role in the conversion of coarse emulsion to nanoemulsion.

### 4.2. Pesticide Nanoemulsion Based on Oil Palm Derivatives

The fruit part of the palm tree has driven the production of palm oil. Palm oil is known as the most versatile vegetable oil as it is rich with vitamin E and balances the saturated and unsaturated fatty acid composition. The use of palm oil as the oil phase in pesticide nanoemulsion has contributed promising stability results. The combination of palm kernel oil ester (PKOE) with *Parthenium hysterophorus* crude extract (PHCE) has demonstrated its ability to control *Diodia ocimifolia* weeds at a fairly lower concentration, 5 g L^−1^ [73].

Moreover, palm oil has also been utilized as a surfactant in nanoemulsion preparation such as methyl ester sulfonate (MES), polyethylene glycol-monooleate (PMO) or polyethylene glycol-dioleate (PDO). This palm oil-based surfactant is quite stable [74] and low environmental risk and showing a low concentration value of residue parameter in the fungicide formulations [32].

A palm-based solvent such as fatty acid methyl ester (FAME) or the so-called biodiesel was found to be a green-friendly alternative solvent due to its lower volatility, toxicity and biodegradability [75]. This solvent is superior for pesticide nanoemulsions application as its high ability to improve emulsifier, active ingredient solubility, viscosity and spray-ability [76]. Previous studies have been conducted using FAME as a solvent in controlling weeds [77,78,79]. Another interesting carrier solvent from oil palm is introduced as palm methyl ester (PME). The PME-based formulation has been more practical for agricultural practice when it comes to cost and availability. It was found that PME can reduce the application dose due to its efficient delivery [76]. The development of PME-based nanoemulsions has shown great potential as an insecticide [80] and molluscicides [81].

The demand for palm oil has risen every year due to its versatile properties which can be altered in many ways to form various products. However, the disastrous disease, the so-called basal stem rot (BSR) disease caused by the *Ganoderma boninense* fungus has affected both the quality and yield of the palm oil. Previously, the researchers have identified the capability of nanoemulsions to treat this kind of plant disease. Phenazine extract nanoemulsions and trichodermin nanoemulsions have effectively inhibited the growth of the fungus by 70.74% [82] and 80.74%, respectively [83].

## 5. Optimization and Characterization of Nanoemulsion-Based Agrochemicals

During the preparation of nanoemulsion-based agrochemicals, the samples must undergo two important steps: preformulation and parametric optimization. After the sample has passed the initial screening, the samples will then be chosen for further chemical and biologic characterizations.

### 5.1. Characterizations for Preformulation

During the preformulation process, the value of HLB or critical micelle concentration (CMC) of surfactants is important to be obtained. These parameters are crucial to determine the starting point for the optimization process. The HLB value would be an indicator of the types of nanoemulsion produced either O/W, W/O or other types. CMC value is a point at which self-assembly occurs and the addition of surfactant after this point is no longer reduces the surface tension. Several techniques have been used in revealing the CMC value such as fluorescence probe [84], surface plasmon resonance [85], dye solubilization [86], tensiometer [87], viscometry [88], calorimetry [89] and electrical conductivity [90].

According to previous studies, the pyrene fluorescence probe is one of the most convenient and precise methods in obtaining CMC value. The absorbance of solubilized pyrene in systems is measured based on the peak difference method. A graph concentration versus absorbance is plotted in which the intersection point of two line segments is referred to as CMC value [91]. However, it was found that this technique is not suitable to determine a system that has low CMC value as the minimum concentration of pyrene solution must be at least 6 × 10^−6^ M to get good pyrene fluorescent spectra. Therefore, a new method called “diluting-concentration” has been introduced as an alternative way for a system with very low CMC value [92]. CMC measurement also affected by the selection of probe molecules due to their charge and functional groups [93].

During the optimizations process, a range of nanoemulsions with different parameters are developed and undergo initial screening process including centrifugation assay, freeze–thaw cycle, heating cooling test and nanoemulsion stability at room temperature, 25 °C. Nanoemulsion with maximum stability in which the phase separation does not occur along a certain period will be selected for further characterizations. This stage is very important in determining their thermodynamic stability studies

### 5.2. Characterizations for Nanoemulsion-Based Agrochemical Formulations

#### 5.2.1. Particle Size and Its Distribution

Dynamic Light scattering (DLS) is the most common technique to determine the size and distribution of nanoemulsion. The small size of nanoemulsion is desirable in achieving optimal efficiency. Samples are diluted with deionized water before the analysis to prevent the multiple scattering caused by aggregation phenomenon which occurs via electrostatic interaction. During the measurement process, the polydispersity index (PDI) is also a concern, as PDI value indicates the system stability. The selection of PDI value which is less than 0.5 is acceptable for agricultural use and is considered as a good uniformity of the particle diameter. The samples with higher PDI will be discarded as they showing polydisperse property which is not suitable to be characterized using the DLS measurement [31].

It was known that size of nanoemulsion was affected by several factors. Many researchers have reported that the size of nanoemulsion is greatly affected by surfactant concentration and its packing parameters. The surfactant packing parameters is also crucial as it may cause changes of surfactant curvature thus imparting finer nanoemulsion droplet [94]. The pecking order of the surfactant is highly related to the ratio of hydrophobic and hydrophilic regions. The surfactant arrangement at O/W boundary with low interfacial tension has created a bicontinuous microemulsion in turn impart smaller particles. Most of the research studies claimed that the increasing ratio of surfactant to oil can make the droplet size small [44].

#### 5.2.2. Viscosity, Zeta and pH Measurement

Electrophoretic properties—or the so-called zeta potential of nanoemulsion—are measured using Zetasizer equipment. The surface properties around the particle determine the zeta as well as pH value as an indicator of nanoemulsion stability. A negative zeta value induces repulsive forces that are greater than the attraction forces among droplets, thus averting the coagulation and coalescence to occur in disperse emulsion. Increasing the oil concentration in a system may contribute to the decreased stability of nanoemulsion [95]. Most pesticide nanoemulsions show alkaline properties with a pH value of 5–6 [96,97]. An Ostwald viscometer has been used to measure the viscosity value of nanoemulsion. The viscosity value may be affected by the nature of surfactants, organic phase components and oil viscosity. Pesticide nanoemulsion produces low viscosity as it is categorized as O/W type with high water loading. However, the viscosity of nanoemulsion can be altered by surfactant concentration [60,96].

#### 5.2.3. Morphology and Stability Study

The shape and morphology of nanoemulsions can be determined using an atomic force microscope (AFM), transmission electron microscopy (TEM), cryogenic-field emission scanning electron microscopy (Cryo-FESEM). Common shapes that have been reported for pesticide nanoemulsions are spherical [98,99] or core shell-like structures [100] due to some cluster of nanomicelles that formed during the preparation process.

Stability tests of nanoemulsions can be conducted by varying the storage time or temperatures. These study are commonly performed at 0, 5, 10 days, but can be over 12 months with the tested temperature at 4–54 °C. During the storage time, the study is accomplished by observing the sample appearance or measuring their physicochemical properties such as zeta potential or particle size at predetermined interval times. Then the sample without any changes in their appearance likes phase separation, creaming, flocculation, coalescence and sedimentation is considered as a stable system. The zeta potential and particle size of the nanoemulsion is measured and compared before and after the storage. The samples with maintain value are assumed in a stable condition.

**Figure 3:**
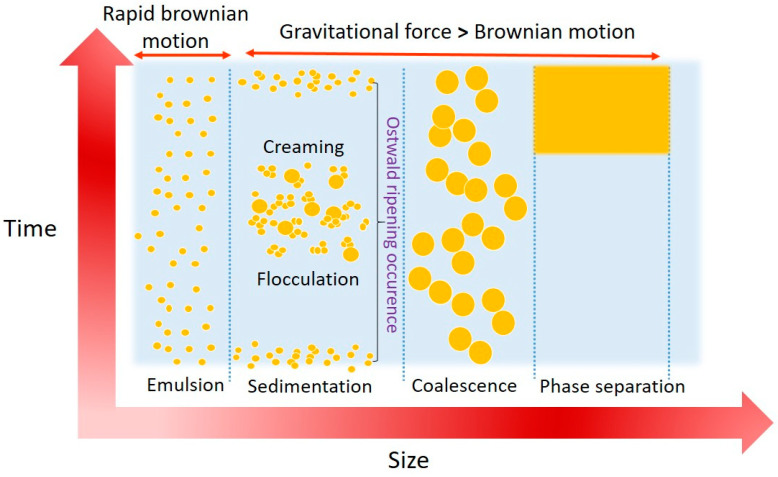
Increase of nanoemulsion droplet size due to instability phenomenon as a function of storage time.

The study also suggested that high temperature can lead to nanoemulsion instability which may cause by the particle movement and dissolution of emulsifier into water impart to the aggregation of particles in emulsion [101]. Nanoemulsion stability also influenced by other factors such as Ostwald ripening which usually occurs at the first 5–10 days after the preparation and then flocculation and coalescence will take place at a later stage, as shown in Figure 3. Ostwald ripening is dependent on the oil phase fraction in the nanoemulsion system. The coarsening mechanism in nanoemulsion stabilized with Brij 30 or mixture of Span 80 and Tween-80 has explained that Ostwald ripening is the main cause of coarsening phenomenon in nanoemulsion with low oil phase fraction which up to 0.05 [102].

All factors other than Ostwald-ripening can be controlled by the appropriate surfactants and small droplet size. High curvature of nanoemulsion obviates the flocculation and coalescence to occur as Laplace pressure prevents the deformation of larger droplets. Ostwald ripening becomes the main concern as it may occur after long term storage which affects during the application. This phenomenon is associated with the conversion of small droplets into larger droplets with low curvature radius which resulting in larger particle size. However, this occurrence can be avoided by increasing the elasticity of droplet [103] and the addition of surfactant which reduces interfacial free energy forming a mechanical barrier against coalescence.

#### 5.2.4. Retention and Contact Angle Measurement

Retention and contact angle of leaves are measured to relate the affinity of the pesticide liquid towards the leaf surfaces. The efficiency of pesticide nanoemulsion can be enhanced by increasing the adhesion work of nanoemulsion towards the leaves. Retention can be measured by dipping and micro weighing method while contact angle of leaves is evaluated using a precision contact angle measuring instrument equipped with a charged coupled device, CCD camera. It worth noted that the contact angle of nanoemulsion decreases as the increasing agrochemical content, showing that active ingredient has low interfacial tension which effectively allowing the pesticide diffusion in the plant surface [104].

#### 5.2.5. Biologic Study

Pesticide nanoemulsions can be tested on their efficiency towards pathogenic organisms. Researchers have come up with different approaches in controlling and killing these pathogens—bacteria, fungi and insects, because they damage plants, thus reduce the quality and plantation yield.

**Figure 4:**
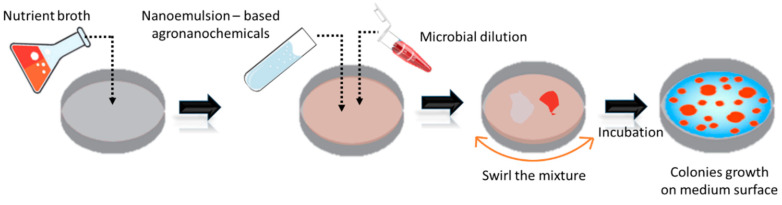
Schematic representation of the antimicrobial activity test using the agar diffusion method.

The common method used for antibacterial and antifungal studies using pesticide nanoemulsion is broth microdilution and agar dilution assay, as described in Figure 4. A study has highlighted that preparation of cinnamon essential oil (CEO) nanoemulsion has greater inhibition zone against *Aspergillus niger, Rhizopus arrhizus, Penicillium sp. and Colletotrichum gloeosporioides* when compared to CEO coarse emulsion [105]. A peppermint oil-based nanoemulsion has been reported as a potential nano pesticide as it reduced biomass of *Alternaria solani* which causes early blight in tomato plants [106]. The nanoemulsion with neem and citronella oil as the actives has decreased the effective dose (ED_50_) towards *Rhizoctonia solani and Sclerotium rolfsii* [98]. Garlic oil nanoemulsion has destroyed proteins in *Penicillin italicum* with lower minimum inhibitory concentration, MIC value of 0.23%, lower than pure garlic oil with value is up to 3.7% [107].

Previous studies have developed nanoemulsion containing a microbe for treating crop disease. Incorporation of biologic fungus, *Talaromyces flavus* in nanoemulsion has shown to inhibit *Fusarium oxysporium* species [108]. *Trichoderma* species have also been encapsulated in nanoemulsion to control downy mildew diseases [109]. It was also noticed that the resistance towards microbial diseases such as *Erwinia carotovora* bacteria, *Aspergillus niger*, *Rhizopus stolonifer* fungus [110] and *Colletotrichum* fungi [62] was increased with the addition of polymer in the nanoemulsion. The finding has mentioned that chitosan nanoemulsion showing better results with lower EC_50_ in combating those plant pathogens.

Some nanoemulsions also were used for controlling unwanted plants such as weed and grass, as these plants compete with the crops for space and essential nutrients. Since these plants are smaller, it is more susceptible than the larger plants. The inhibition by *Thymus capitatus* and *Majorana hortensis* nanoemulsion towards bindweeds have shown that the nanoemulsion has suppressed the weed growth by reducing more than 50% of the fresh and dry weight of both *Convolvulus arvensis* and *Setaria viridis* than the control [111]. Nanoemulsion-derived palm oil derivatives used as a pretreatment of slender buttonweed, *Diodia ocimifolia* seeds also indicates that it has toxicity effect towards germination and capable to inhibit at the lowest concentration of 5 g L^−1^ [73].

Recent studies have investigated the enhancement of toxicity effect of pesticide nanoemulsions towards the targeted pests. *Mentha longifolia* nanoemulsion display slow release of essential oil and longer contact toxicity against *Ephestia kuehniella* larva [112]. Span 80 and Arabic gum as surfactants in Tasmanian blue gum essential oil nanoemulsion has indicated higher toxicity and ovicidal effect towards *Callosobruchus maculatus* adults [113]. Garlic essential oil nanoemulsion that uses water as a solvent has shown good acaricidal activity towards eriophyid olive mites with no toxicity effects towards the albumin and total protein in rats [114].

Other investigations have shown the evidence of morphologic and histological damages of insects after nanoemulsions treatment. They have examined the capability of nanoemulsions to penetrate the insect cuticle and cause serious injury and irritations to the insect pests. SEM investigation has shown that the *Pimpinella anisum* essential oil nanoemulsions have caused necrosis and blackening on *Tribolium castaneum* [98]. The cuticle of the insect was severely damaged, resulting in no difference was seen between the exocuticle and endocuticle. The regenerative cells also recorded fewer compared with the control, leading to dilation of internal structure which causes death. The author has reported a mixture containing soybean oil, tri-n-butyl phosphate and Triton X–100, BCTP nanoemulsion can treat *Bacillus spore* in rats by 98%. After exposure to nanoemulsion, inflammation and inflation of cellular structure in the rat have been observed which better than untreated ones, which showing serious tissue necrosis [115].

Nanoemulsions were also evaluated as promoting growth, as it can give significant effects towards the plant seedling either as a promoting growth or seed treatment. It was found that the nanoemulsion derived from thymol essential oil and saponin was able to suppress bacterial growth while promoting soybean plant growth [71]. Eugenol oil nanoemulsion enhanced the germination process and produced high resistance toward the fusarium wilt disease on the cottonseed plant [116]. Methylcellulose nanoemulsion as a seed invigorate has improved the growth of maize seedling by increasing the root and shoot length by 18% and 33%, respectively [100]. Seed priming with turmeric nanoemulsion has resulted in better germination growth of watermelons [117]. The combination of pectin with neem oil nanoemulsion exhibit growth promotion to the soybean seeds [118].

## 6. In Vitro and In Vivo Study of Nanoemulsion-Based Agrochemical Formulations

Oil in water nanoemulsion was used as a carrier to deliver agrochemicals for agricultural applications. The development of nanoemulsion-based agrochemicals has become a method to overcome the water-insoluble pesticides [119]. Table 3 presents a list of patents on nanoemulsion-based agrochemicals used for various purposes.

Few studies have highlighted the use of various polymers as carriers in the preparation of various controlled-release agrochemical nanoemulsions. A biodegradable castor oil polyurethane loaded with avermectin and λ-cyhalothrin has been designed and their release property from the carrier has been studied. A study has shown that both acid and alkali medium accelerates the avermectin release to almost 100% compared to only 72% for the control at the neutral condition [104]. They identified that the release rate of λ-cyhalothrin nanoemulsion is much slower which was lasted for 180 h compared to 60 h for a commercial product [135]. Both studies also stated that the cumulated release of agrochemical increased when the sample has high agrochemical content and at elevated temperature. Emamectin benzoate has been synthesized using polymeric nanocapsules (PNC) and two different nanosilica were used as the carrier. The study demonstrated that a maximum cumulative release of 51% was found in EB loaded with silicon dioxide nanoparticles (SNPs) [136]. This nanoformulations also protect some agrochemical compounds from photodegradation using the coating technique. They have coated deltamethrin using double layers composed of chitosan and lignosulfonate. High ability of coating materials to absorb UV radiation has reduced the effect of photosensitizers towards deltamethrin by half and the thickness of the layer has induced slow release of deltamethrin from the system [63].

**Figure 5:**
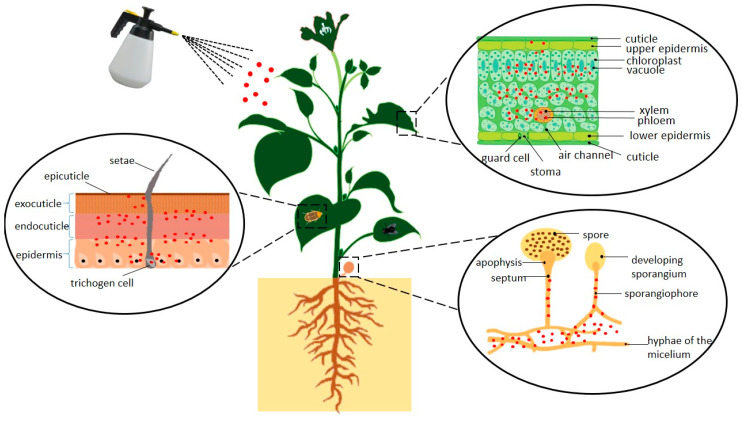
Schematic diagram showing the penetration of nanoemulsion-based agrochemicals through the leaves surface, insect cuticles and fungal body.

The generation of nanoemulsion-based agrochemicals has enhanced the biologic properties by improving bioavailability and cell uptake of the agrochemicals. The diffusion of agrochemicals into the plant cuticle, insect cuticle and microorganism structure is schematically illustrated in Figure 5. Better absorption of active ingredient has created efficient diffusion through the plant cuticle and produce good wettability. A study has reported that avermectin nanoemulsion has increased the spreading ability of cabbage and cucumber leaves with the value of contact angle of 46–58° compared to 79–106° for water [101].

This innovation has boosted the lipophilicity properties allowing successful impregnation of nanoemulsions through the insect cuticles. This phenomenon causes a deep effect on many parts of body insects thus increase the efficient uptake of agrochemical as an active ingredient. This penetration will disrupt the wax cuticular layer on the insect, impart to rapid water loss and finally causes mortality of insects. They have investigated the potential of azadirachtin for controlling pests in crop plantations. It was identified that the nanoemulsion with small particle size exhibited better toxicity towards adults *Sitophilus oryzae* and *Tribolium castaneum* compared to the control [137,138]. Bifenthrin nanoemulsions have also increased the mortality rate of cabbage maggots to 100% after 20 h of exposure time [139]. A microemulsion based on norcantharidin [140] and emamectin benzoate [19] has been formulated to control diamondback moth. Other nanoemulsions have been developed to containing organophosphorus and pyrethroid insecticides, and they also have shown a better effect than their counterparts, free insecticides [97,141].

The incorporation of herbicides in nanoemulsion systems is expected to enhance the penetration of herbicides into plant tissue, thus facilitating good weed control with low herbicide dosage. Glyphosate isopropylamine (IPA) has been used as an active ingredient for the preparation of herbicide nanoemulsion [112] and was found to promote higher herbicidal efficacy towards weed such as *D. ocimifolia, P. conjugatum, A. gangetica* [78] and *Eleusine indica* [30].

**Figure 6:**
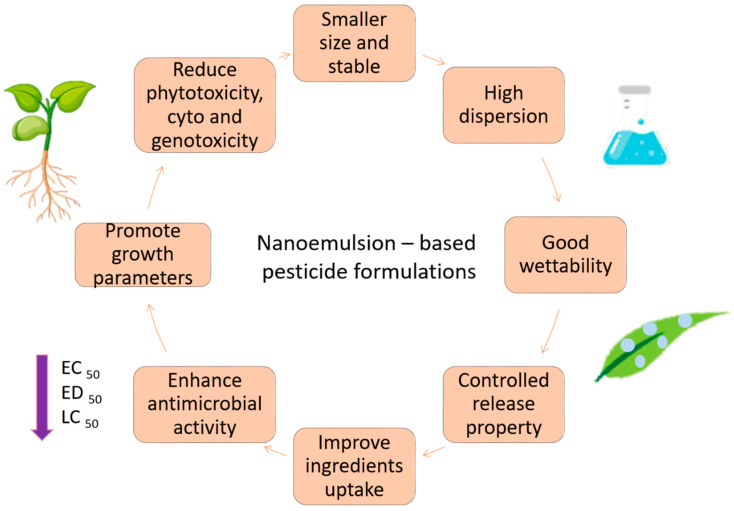
Flow diagram showing the fabrication of nanoemulsion–based pesticide formulations that have improved the physicochemical and biologic activities.

Fungicide-based nanoemulsions are capable of reaching the fungal cell membrane due to their small size. The formulation of the nanoemulsion increases the water solubility of fungicides, allowing the fusion with phospholipid bilayer of microorganisms and accessing the cell membrane surface. This permeation facilitates the breakdown of cells, followed by cell death. Tebuconazole nanoemulsion has been fabricated as a potential fungicide with low toxicity compared to its counterpart, the commercial products [31]. The same is true for nanoemulsions containing mancozeb; these have shown better efficiency on antifungal activity towards *Glomerella cingulate* [54]. The above-mentioned capabilities of nanoformulations have been resulted from their superior physicochemical and biological activities due to their nano-sized, as summarized in Figure 6. 

## 7. Environmental Risk Assessment of Nanoemulsion-Based Agrochemicals

Great interest has been raised about how nanopesticide products—including nanoemulsion-based agrochemicals—are assessed by their environmental risk assessment (ERA) before they are readily introduced into the market. It has been proposed that formulations development through nanotechnology approaches may affect the toxicological or safety profile systems as the alteration of additives and properties of the systems has occurred. In nanoemulsion systems, the presence of surfactants would contribute a higher risk towards the environment and health compared to the non-modern formulations due to its potentially hazardous [35].

Nowadays, tiered principles have been used in facilitating a harmonized approach for environmental assessment. This tiered approach involves the effect and exposure assessment which lastly resulting in a final evaluation. The process takes four steps: microbial community test, laboratory and modeling, biomagnification and recovery and lastly field monitoring studies. During each step, the comparison between predicted environmental concentration (PEC) and predicted no-effect concentration (PNEC) in soil and freshwater are examined. This principle has fully described elsewhere [142].

Recently, a risk assessment tool for nanopesticides based on computer-based chemical technology using computational method has been developed and known as nano-quantitative structure–activity or structure–property relationship (nano-QSAR/QSPR). This modeling method can relate physicochemical properties to environmental fate and their degradation products [143]. Generally, these tools take into account both quantitative and qualitative data and generating results using statistical validation. The implementation of the nano-QSAR/QSPR tool will make a better decision for regulatory purposes by providing precise and accurate outcomes for nanoformulation safety and its functionality [144].

## 8. Conclusions

This brief review has shown that nanoemulsions have great potential to develop lipophilic active-loaded products for pesticide applications. Previous work has shown that the use of nanoemulsion as a carrier of pesticides has tremendous advantages. In addition, the nanoencapsulation approach could improve the physicochemical properties and stability by enabling their water dispersibility, reducing their volatility and protecting them from the external environment.

The tunable size and stability of nanoemulsion-based agrochemicals are controlled by the nature of the constituent components and their concentration for the formation of the nanoemulsion systems. It was suggested that the modification of nanoemulsions to smaller size has influenced the effectiveness of the antimicrobial activity towards microbial pathogens. Most of the studies have shown that the antifungal or insecticidal activities were more efficient in their nanoformulations form, in which generally the smaller the size the better, as the components or molecules entrapped were optimum and the surface area is high.

## 9. Way Forward

Apart from other agrochemicals, nanoemulsion-based pesticides show many advantages compared to traditional pesticides. These nanoformulations show excellent properties that reveal the positive impacts on their biologic efficacy. The proper application of nanoformulations can trigger the release of active ingredients to the targeted area in a controlled manner.

Although water-soluble pesticides are desired in agricultural applications, the increase in solubility is not always synonymous with safety. This solubility property may potentially contaminate water sources. Therefore, it was suggested that nanoemulsion-based agrochemicals should be tested further for ecotoxicity studies as a risk assessment to ensuring the level of solubility that causes harm to living organisms.

Previous findings have focused more on in vitro studies—especially the antimicrobial activities—but in vivo studies are still lacking. Although phytotoxicity studies on the germination of the seedlings have been conducted, unfortunately, more effort and studies should be made to convince and establish them in real applications and the market.

Systematic studies are required for actual field investigations using mature plants to examine the effectiveness of pesticide nanoemulsions for better applications. Studies on various plant diseases treated with nanoemulsion-based agrochemicals are based on their physiological parameters. There is a need to identify the relationship of real plantation practices with nanoemulsion capabilities. The stability of nanoemulsion-based agrochemicals is another challenge, as pesticides are environmentally responsive. The resistance of pesticide nanoemulsions to the environmental factors such as temperature and pH for prolonging storage time and preserving active ingredients needs to be reviewed. More selective pesticide nanoemulsions must be developed to reduce the effects on organisms. The cytotoxic and genotoxic effects of pesticide nanoemulsions towards animal cells have been addressed, but the effect on human cells should be further investigated. These may lead to knowledge-generation and contributions—and subsequently tap the good potential of nanoemulsion-based agrochemicals as the future generation of safe nanopesticides.

## Figures and Tables

**Table 1:** Example of single and mixed surfactants used for the preparation of pesticide nanoemulsion.

Type of Compound	Compound/Name	Conditions	Mean Size (nm)	References
Anionic	Polysorbate 80 (Tween 80)	Mixed with organic phase, stirring at 2400 rpm for 20 min	<100	[24]
	Polysorbate 80 (Tween-80) and sorbitan monooleate (Span 80)	Mixed with organic phase and stirred at 400 rpm for 30 min under controlled temperature, 80 ± 5 °C	Below 500	[25]
	Polysorbate 20	Mixed with organic phase and undergo sonication process	99–170	[26]
	(Tween-20)	Mixed with oil phase at 45 °C	125–334	[27]
	Montanov 82	Mixed with aqueous phase	50–150	[28]
	Polyoxyethylene nonyl phenyl ether (TX-10)	Mixed with aqueous phase	200–400	[29]
	Green polyoxyethylene glycerol fatty acid ester or glycereth-17 cocoate	Mixed with organic phase under stirring	41–100	[30]
Nonionic and anionic	Agnique BL1754 (AG54)	Mixed with organic phase, stirring at 400 rpm for 30 min at 40 °C	250	[31]
	Palm oil-based. anionic: methyl ester sulfonate (MES)/Nonionic: polyethylene glycol dioleate (PDO)/and polyethylene glycol monooleate (PMO)	Mixed with oil phase under 1000 rpm for two hours at room temperature	350–480	[32]
	Polyethylene glycol dioleate (nonionic) and toximol (ionic)	Mixing with oil phase, vortexes at room temperature	5–20	[33]

**Table 2:** Examples of oil used as the organic phase in the pesticide nanoemulsions.

Dispersion Composition	Optimal Conditions	Purpose	References
Carvone, cinnamaldehyde, citral, geraniol, pulegone oils (5 v/v%)	Sonication at 75 kHz, 9 cycle/s for 15 min	Antibacterial activity against *Pectobacterium carotovorum* and *Ralstonia solanacearum*	[46]
Neem oil (0.5–3 w/v%)	20 kHz, 9 cycle/min for 10 min	Antifungal activity against *Aspergillus flavus* and *Penicillium citrinum*	[47]
Sweet basil, marjoram, peppermint, spearmint and thyme essential oils (10 v/v%)	Sonicated at 700 W for 30 min	Antifungal activity against *Fusarium oxysporum*	[48]
Thyme essential oil (10 v/v%)	Stirring at 700 rpm for 15–60 min	Antifungal activity against *Sclerotinia sclerotiorum*	[49]
Citronella and lemongrass oil (10 v/v%)	Stirring at 700 rpm for 30 min	Antifungal activity against *Botrytis cinerea*	[50]
Sweet flag oil or *Acorus Calamus* (6 v/v%)	Stirring at 700 rpm for 2 h	Insecticidal activity against rice weevil, *Sitophilus oryzae*	[51,52]
	Sonicate at 1500 W for 30 min	Insecticidal activity against pulse beetle	
Clove oil (1 w/v%)	Kept under moderate stirring for 10 min	Antifungal/cytotoxicity activity against *Glomerella cingulata*	[53]
		Ecotoxicity and genotoxicity effect against *Folsomia candida*	
Eugenol oil (3.3 v/v%)	Homogenized at RT	Antifungal activity against *Glomerella cingulata*	[54]
Vegetable oil and fatty acid methyl ester (1.9–7.5 w/w%)	Stirred at 200 rpm for 5 min	Herbicidal activity against *Eleusine indica*	[30]
Clove and lemongrass oil (5 w/w%)	Stirred at 750 rpm for 30 min	Antifungal activity against *Fusarium oxysporum*	[55]
*Eucalyptus* oil (6–10 w/w%)	Stirred at 750 rpm for 60 min Homogenized at 5000–20,000 rpm for 2–20 min	Insecticidal activity against *Tribolium castaneum* and *Sitophilus oryzae*	[56,57]
*Rosmarinus officinalis* oil (5 w/w%)	The aqueous phase was added dropwise into oil phase at flow rate 3.5 mL/min, stirred at 800 rpm for 60 min	Larvicidal activity against *Aedes aegypti*	[58]
Castor oil (1.38 w/v%)	Preformulation is heating to 80 °C under stirring at 300 rpm and cooling process at RT. After neutralization, the final mixture was stirred at 1100 rpm for 40 min	Pesticide applications	[28]
Linseed oil (10.5 w/v%)	Preformulation undergoes two heating stages, at 180 and 235 °C under stirring speed of 300 rpm for the total 3 h. The mixture is cooled at a temperature of 150 °C and heated again at 175 °C before neutralization process. Final mixture was stirred at 1600 rpm for 40 min		
Cinnamon oil, thyme oil, manuka oil and tea tree oil (1%–3%)	Adding the aqueous phase into oil phase, drop-by-drop and continuous stirring at 500 rpm at RT. Directly mixed with aqueous phase and undergo sonication of 50% amplitude at 20 W for 1 min	Antimicrobial activity	[59]

**Table 3:** Patents on nanoemulsion-based agrochemicals for agricultural applications.

Patent/Year	Method of Preparation	Purpose of Invention	Reference
CN108967422A/2018 emamectin-benzoate nanoemulsion and preparation method thereof	A mixture of emamectin benzoate, botanical solvent (palm oil, castor oil, soybean oil or more), polar solvent (tertbutyl alcohol, isopropanol or more), surfactant (Tween-80, polyoxyethylene polyoxypropylene block type polyethers, alkylphenol-polyethenoxy or more), deionized water and surplus	The product showed a nanoemulsion size of 20–200 nm The nanoemulsion can be used to control diamondback moth.	[120]
CN108935449A/2018 Nanometer biphenthrin emulsion in water and preparation method thereof, field fly the purposes of anti-application	Mixing of biphenthrin, dimethylbenzene, monododecylphosphate potassium, fatty alcohol polyoxyethylene ether AE0-3, ethylene glycol and water surplus	The product is used against flies in field applications. The nanoemulsion has high stability after 5 and more times dilution.	[121]
WO2017033153A1/2017 oxadiazon nanoemulsions	An oxadiazon nanoemulsion comprising oxadiazon active ingredient, organic solvent with alcohol group (acetone, cyclopentanone or more), non-ionic surfactants containing polyalkylene glycol, aliphatic alcohol (butyl alcohol, ethyl alcohol or more) and water	The product enhanced the herbicidal activity towards grass weeds and broadleaf weeds in pre- and post-emergence of crops in rice and corn plantation. The efficiency of nanoemulsion achieved more than 58% with high selectivity properties as the invention does not affect the rice cultivation throughout the treatment process.	[122]
CN106489989A/2016 A kind of nanoemulsion insecticide and preparation method thereof	A mixture of λ-cyhalothrin, pleocidin, matrine, nano titanium oxide, soybean oil, egg yolk lecithin, n-butanol, Tween-80, avermectin and griseofulvin The insecticide nanoemulsion was prepared using ultrasonication method	The product is used as an insecticide for field application.	[123]
CN106577684A/2016 Pesticide nanoemulsion in water and preparing method thereof	The mixture consists of pethoxamid, garagard, solvent (d-limonene, repefral or propene carbonate), polyoxyethylene emulsifying agent, anti-freezing agent, thickener, defoamer and deionized water Phase inversion temperature was chosen as a method of preparation	The product produced nanoemulsion with a size of 300 nm and has high stability. It is beneficial in controlling broadleaf, grassy weeds of a soybean field and smothering soybean plantation Promote the soybean growth and has the potential to increase production yield.	[124]
CN105994345A/2016 Efficient cyfluthrin nanoemulsion composition and preparation method thereof	A composition comprising betacyfluthrin, liquid white beeswax, Span-80, Tween-80 and excess water	Nanoemulsion size is 69–150 nm and is used as a pesticide in field application.	[125]
CN105052902B/2015 Slow controlled-release pesticide nanoemulsion and preparation method thereof	A mixture of water-insoluble agrochemicals such as avermectins and λ-cyhalothrin and polyurethane as a carrier	The product produces slow/controlled release pesticide with good dispersion, bioavailability and stability.	[126]
CN104206406A/2014 Nanoemulsion pesticide and preparation method thereof	A composition comprising cyhalothrin, pleocidin, matrine, nano titanium oxide, soybean oil, egg yolk lecithin, n-butanol and tween-80. The nanoemulsion has been prepared using ultrasonication method at 200–400 W within 5–15 min	The product has a size in the range of 60–80 nm Nanoemulsion reduced the median lethal concentration (LC_50_) of diamondback moth larvae.	[127]
CN103461360A/2013 Preparation method of avermectin-griseofulvin composite nanoemulsion pesticide	A mixture of avermectin, griseofulvin, oil phase (ethyl oleate, soybean oil or atoleine), emulsifier (albumin, lecithin, stearic acid or more) and emulsifying agent (ethylene glycol, ethanol, propane diols or more)	The product has improved insecticidal activity of the nanoemulsion towards diamondback moth, resulting in a low value of LC_50_ and stable up to 3 months.	[128]
CN109452269A/2013 Avermectin emulsion formulations and preparation method and application	A mixture of avermectin, organic solvent (methylene dicarbamate and propylene-glycol diacetate), emulsifier (tristyrylphenol polyoxyethylene ether, phenethyl phenol polyoxyethylene poly-oxygen propylene ether or more), stabilizer (2, 6-toluene di-tert-butyl phenols (BHT), butylhydroxy anisole (BHA) or more) and water	The product has a good killing effect towards lepidoptera larvae of brassicaceous vegetables and the efficiency of nanoemulsion can be reached up to 60% towards wild cabbage *Plutella xylostella* and cotton red spider. The nanoemulsion also can be used to prevent and treats cucumber root-knot nematode.	[129]
CN102599186A/2012 Efficient cypermethrin nano pesticide emulsion	A mixture of a non-ionic surface active agent (Triton X-100), anionic surfactant (lauryl sodium sulfate), cosurfactant and cypermethrin Constructed by spontaneous emulsification method	The average size of the nanoemulsion is 11 nm The product has a better killing effect on insects with low environmental pollution.	[130]
CN102919226A/2012 Abscisic acid nanoemulsion and preparation method thereof	A mixture of abscisic acid oil phase (carrene or dimethyl sulfoxide), polyvinyl alcohol–water solution, emulsifier (lauryl sodium sulfate, dodecyl sulfobetaine, alkyl dimethyl sulfoethyl betaine, Tween-80 or more) and stabilizing agent (xanthans)	The product produced nanoemulsion with a slow release of abscisic acid which prolongs the action time leading to a better novel agrochemical application system. The particle size distribution of nanoemulsion is between 10–100 nm.	[131]
CN103210952B/2012 Methidathion nanoemulsion and preparation method thereof	A composition comprising methidathion, toluene as an organic solvent, emulsifier (Arlacel-60, Arlacel-80, Tween-60 and Tween-80), co-solvent (PEG-200, PEG-400 or PEG-600), antifreeze (one or any combination of ethylene glycol, propane diols or glycerin), water, thickener (two stearates of hydroxyethyl cellulose, methyl hydroxyethyl cellulose, ethyl hydroxyethyl cellulose or more) and antifoaming agent silicone defoaming agent	The product produces methidation nanoemulsion as a pesticide with low toxicity and good biodegradability and enhanced stability.	[132]
CN102960337B/2012 Butralin nanoemulsion and preparation method thereof	The composition comprising butralin oil phase (dimethyl sulfoxide), polyvinyl alcohol–water solution, emulsifier (lauryl sodium sulfate, cetane trimethyl ammonium bromide, cetyl pyridinium bromide or more) and stabilizing agent (xanthans, bentonite, sodium alginate or more) The nanoemulsion was prepared using ultrasonication method.	The product has good stability for up to 12 months and able to suppress tobacco axillary bud growth.	[133]
WO2011010910A1/2010 A herbicide formulation	Mixing of glyphosate salt (any one or combination of glyphosate isopropylamine and glyphosate ammonium) as an aqueous phase, oil phase of methyl ester mixture and alkylpolyglucosides and an alkyl organosilicon surfactant systems	As an herbicide formulation to destroy the targeted plant Possesses good stability for long term release of the glyphosate active.	[134]
